# Effect of transport infrastructure development on selected components of the environment of inner-city river valley and the possibility of its revitalization (Lublin, Poland)

**DOI:** 10.1007/s11356-022-18964-y

**Published:** 2022-02-09

**Authors:** Tomasz Zubala

**Affiliations:** Department of Environmental Engineering and Geodesy, University of Life Sciences in Lublin, Leszczyńskiego Street 7, 20-069 Lublin, Poland

**Keywords:** River valley, Transport, Pollution, Degradation, Revitalization, Urban succession

## Abstract

The study covered an urban river valley, strongly transformed due to the transport infrastructure development. The paper evaluates changes in spatial management of the valley section passing through the city centre that occurred during the past two centuries (long-term and short-term phenomena) as well as their effect on selected environmental components. The basic spatial analyses were carried out with the use of specialized software, cartographic materials and photographic and descriptive documentation of the studied area (archival data). The most unfavourable changes in the potential of the valley environment occurred over the past few decades. They are manifested in a considerable deterioration of landscape values as well as the quantity and quality of water resources. Relationship was identified between water quality and intensity of motor vehicle traffic near the river. Despite the progressing urbanisation of the valley, revitalisation procedures can be still carried out and attractive space in the city centre can be created. In order to demonstrate the reasonableness of the presented concept, the paper suggests that *urban succession* should be incorporated as a term covering time trends and accumulated transformations. It can be helpful in analysing and determining the directions of development in disputable situations.

## Introduction

The condition for permanent sustainable development on a local and global scale is to maintain the order and balance between the potentials of the environment, society and the economy. This is applicable to many different areas and levels of human activity, including transport infrastructure planning (Peng et al. 2020; Zoeteman et al. 2016). Negative consequences of improper management decisions due to, for instance, lack of knowledge, experience, non-compliance with the rules of prevention or choosing “the lesser evil” can be noticeable over decades. The return to relative balance is often long-lasting and expensive. Sometimes the given space must be completely reorganized, taking new functions and management methods into account.

Planning the transport network is one of the more difficult tasks. In countries like Poland, the construction of motorways and expressways was commenced relatively late in comparison to other West European countries (economic barriers). The situation on city ring roads was even worse. Before Poland joined the European Union, such solutions had been virtually non-existent. Transit motor traffic ran through cities, often near their historic centres. This determined how those zones were managed and how the environment vs. society system operated (Ministry of Infrastructure 2020). A considerable area covered by arterial roads and line concentration of multiple pollutants, given the concurrent lack of efficient protective solutions, resulted in transformations of the environment and landscape. The flow and balance of the matter were disturbed (Luo et al. 2015; Virág et al. 2021). This was particularly harmful to river valleys that often constitute an important element of the system of undisturbed exchange of air in urban areas (Chan and Vu 2017; Ren et al. 2018). The then planners of road infrastructure considered flat bottoms, long straight sections enclosed with slopes or close neighbourhood of “cheap” receivers of polluted rainwater exceptionally attractive. The reverse effect of the environment and related possible losses were of secondary significance. With time, cities as complex and dynamic natural and economic systems started exporting more and more products of their activity (e.g. water and air pollution, noise, wastes), having a significant effect on living conditions (Li et al. 2017; White 2002).

Since the late twentieth century, an increase has been observed in the level of awareness and requirements of urban communities regarding the quality of the environment, health-related living conditions and standard of work and leisure conditions. Therefore, in the process of urban planning and transport development, the problem of ecological enrichment of the spatial structure of cities is raised more and more often (Arslan et al. 2020; Suárez et al. 2020). The general interest of society and a broad context of attractive living conditions in the city should be emphasized and careful studies must precede the identification of areas for natural revitalisation (Angelidou and Psaltoglou 2017; Frantzeskaki 2019).

The aim of this paper is to evaluate changes in spatial management of the selected river valley passing through the city centre that occurred during the past two centuries (long-term and short-term phenomena) as well as their effect on the existing environmental conditions. Particular attention was paid to the development of transport infrastructure. The analysis was carried out in the context of general trends in spatial planning in Polish urban areas, using the example of the city of Lublin (south-eastern Poland). Significant changes in the management and use of the inner centre of the city occurred over the past few decades. Some of them have a beneficial effect on transport safety and availability. However, they can, on the other hand, have a negative effect on respective components of the environment, e.g. waters or landscape. It was the reason for, among other things, evaluating changes in the quality of water in the Czechówka River for varying intensity of traffic within the neighbouring main transport artery (before and after the construction of the outer ring road of the city). The article also suggests that corrections should be made to the planning concept if a considerable part of motor traffic is driven out of the city. The purpose of such corrections is to enhance the landscape values of the river valley, protect its water resources and satisfy other needs of society. In support of the reasonableness of the general concept, new terms *urban succession* and *urban climax* were proposed in the article. They covered time trends and accumulated transformations, including phases of more spontaneous space management. They can help analyse and determine the directions of development in disputable situations. The results should be a valuable source material for experts in environmental management, environmental protection, water management and spatial planning and management.

## Material and methods

### General conditions

Lublin is situated in south-eastern Poland near the northern edge of the Lublin Upland. It lies within the moderate continental climate zone. The mean annual ambient temperature is 7.7 °C, and the precipitation total amounts to 598 mm (1971–2010). The lowest mean monthly temperatures and precipitation totals are recorded in January and February, and the highest in July (SP 2018). Lublin is the largest city of eastern Poland and extends over an area of 147 km^2^. It has about 340 thousand inhabitants (SO 2019). Many industrial plants representing various areas of industry operate within the Lublin urban area. Road infrastructure and housing as well as commercial and service building development have been developing dynamically. New districts are built mostly at the outskirts of the city, whereas the central zone is filled with old infrastructure. Revitalisation and modernisation are required in many cases.

**Fig. 1:**
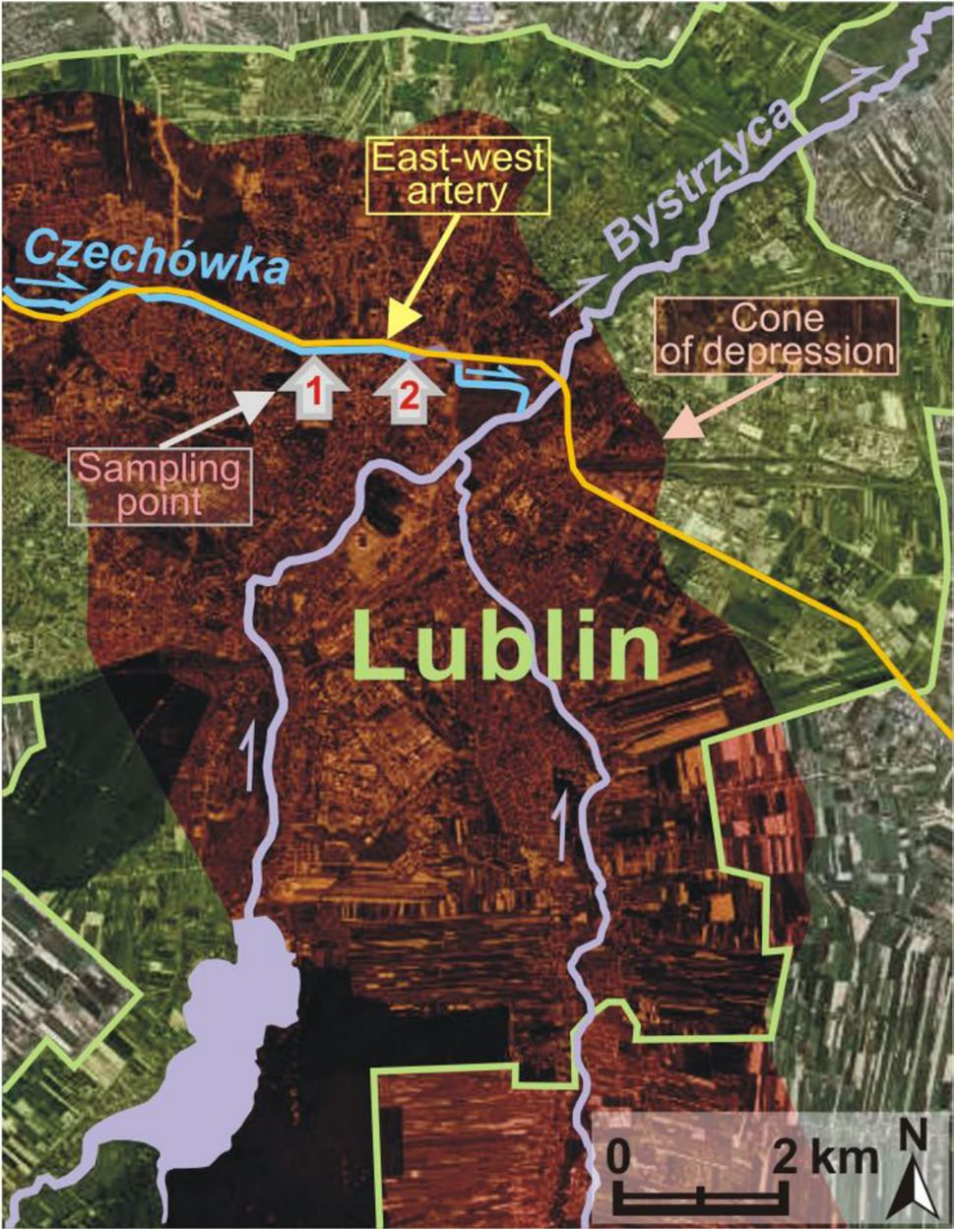
The location of the Czechówka River and checkpoints 1 and 2

The Czechówka River under observation is a left-bank tributary of the Bystrzyca River (Fig. 1) which in turn disembogues into the Wieprz debouching into the Vistula—Poland’s main river. The Czechówka River is 17.5 km long and its catchment basin extends over about 80 km^2^. The whole lies within a loess plateau intersected by gorges and dry valleys. The river head is situated at 227 m above sea level and the estuary is at a lower altitude, i.e. 166 m above sea level. The lower part of the catchment, within which the checkpoints are located, remains under a strong influence of the city.

The Czechówka catchment contains the most essential elements of the city’s natural system—e.g. gardens, parks and allotment (community) gardens. The valley of the river is parallel oriented—from the west to the east. Within the city limits, the valley is from 100 to 300 m wide and it cuts several metres deep into the ground. The valley is well-developed and has a flat bottom filled with sandy silts, alluvial soils and a several metres thick layer of peat. They are underlain by quaternary sand and sand and gravel deposits. In the bottom of the valley, underground water occurs at shallow depths under the surface of the land (Kociuba 2003). The slopes are very steep (left side: 5–18%, right side 5–11%).

### Analysis

The studies were carried out in 2013–2020. In order to evaluate historical changes in the spatial management of the river valley and their environmental effects, a vast body of archival data was collected and compared—including but not limited to digital maps of the city of Lublin from the MAPSTER internet collection of cartographic materials (1:8500—1829, 1:8200—1884, 1:25,000—1943, 1:10,000—2001), photographs from the collection of Grodzka Gate – NN Theatre and old scientific studies. Current natural, landscape and economic conditions were determined based on numerous field observations, analysis of topographic maps, hydrographic maps, land ownership maps and orthophotomaps. Basic spatial analyses were cartometric analyses using the QGIS programme (for instance, placing the maps in a specified system of spatial references). In 2019, geodetic measurements were carried out within the river bed along a 1200-m long section. A GNSS Topcon HiPer V receiver and a Topcon FC-2600 controller were used. Particular attention was paid to the impact of the transport infrastructure on the topography, flora, the location of the river bed and its parameters.

The studies took into account factors that could determine considerable variability of water resources in the Czechówka River. The checkpoints were situated in sections with similar use of adjacent grounds. They are 1100 m apart. The river bed in between them contains 17 rainwater sewerage outlets draining water out of the inner centre of the city, including the transport artery. The analyses of physical and chemical properties of river water were carried out seasonally in 2013–2016. Laboratory tests were performed at the Water and Wastewater Laboratory of the University of Life Sciences in Lublin. Selected physical parameters and oxygen, nutrient and salt content were determined (15 parameters in total). The following devices were used: WTW MPM 2010, WTW Multi 340i and Slandi LF 205. Statistical variability of the results was based on the standard deviation and the coefficient of variation. Thanks to a non-parametric Mann–Whitney test the significance of variation in water quality in the period with high (2013–2014) and low (2015–2016) intensity of motor traffic (before and after the construction of the outer ring road) and in the cold (autumn–winter) and warm (spring–summer) season could be determined. The Wilcoxon test was used to compare water quality indicators in both sections of the river (checkpoints 1 and 2) (Weaver et al. 2017). The analyses were carried out for the level of significance *α* = 0.1.

In 2013–2016, water levels and flow intensity were also measured at checkpoint 2. Measurements were carried out once a month and in the remaining years at water sampling dates. The flow volume was determined by an indirect sectional method after determining the cross-sectional area of the river bed and measurement of the flow rate (floating element).

## Results and discussion

### Historical changes in the development and landscape of the river valley

In the past centuries, changes in the management and use of the analysed river valley resulted from the necessity to satisfy the basic needs of local communities and improve general living conditions which at that time corresponded to the existing potential of civilizational development. These, in turn, always result from the interaction of three systems: natural, social and economic system. Similar to other big cities, an increasingly intensive development of the second and the third system was observed, which was mostly possible thanks to the use of the natural system, that is, at its expense (Chmielewski 2005; Pincetl 2012). With time, the growing disparities in the development of respective systems gave rise to many environmental problems (Tarolli et al. 2014; Xue 2012).

**Fig. 2:**
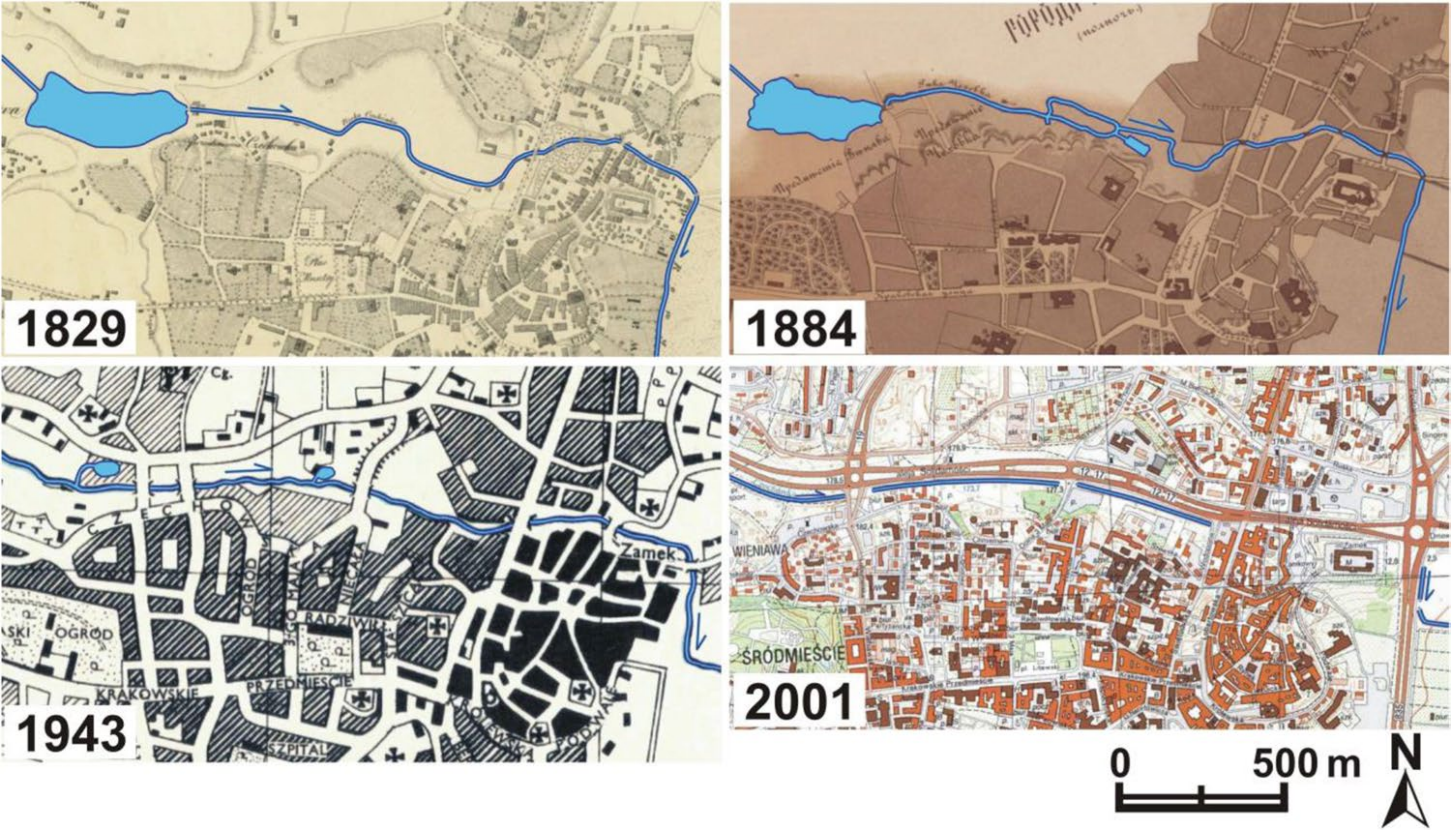
Archival maps presenting the analysed section of the valley of the Czechówka River (MAPSTER 2019)

Changes in the method of management and use of the valley of the Czechówka River in the analysed period must be considered adverse for the natural values of the city. The wide bottom and steep slopes of the valley, almost completely free from technical infrastructure at an early stage, were slowly becoming covered with a network of various categories of roads. Roads in the city centre developed along with roads connecting this part of the city with its northern perimeter. The target undertaking was the construction of a two-lane transit road along the whole length of the valley. As a result, traffic could be diverted from a narrow winding road used for centuries, located along the northern slope. For this reason, the natural river bed reaching 5 m in width and meandering across the whole valley was mostly transformed into a straight, deep-cut drainage ditch running on the right side of the valley (Fig. 2).

Water reservoirs built alongside the river—visible on the maps from 1829, 1884 and 1943—were gradually transformed and eliminated. The same applied to wet meadows (partially flooded in spring) and agricultural fields. At the foot of the slopes in the loess, mother rock vertical escarpments of considerable height were formed naturally. Their location can be seen on the maps from 1829 (north and south sides) and 1884 (south side). The escarpments were also eliminated in the process of building development of the valley, thereby depriving the loess landscape of its characteristic land forms. A thick layer of organic soils in the bottom of the valley was strongly transformed, which is an effect of the drying out of peat and muck formation and displacement of native—and introduction of foreign—soil masses (Kociuba 2003). Apart from the road infrastructure, the analysed territory was gradually covered by housing development, service and commercial buildings.

**Fig. 3:**
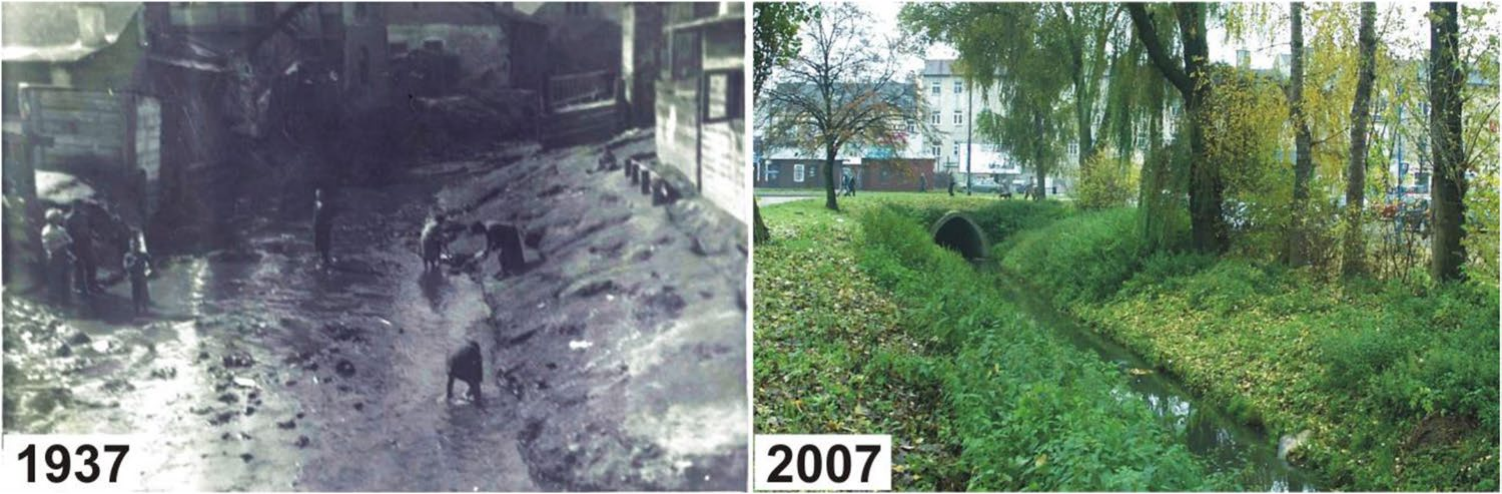
Fragments of the downstream section of the Czechówka River in 1937 (Grodzka Gate – NN Theatre 2019) and 2007 (the concrete vault of the river bed is visible in the background)

**Fig. 4:**
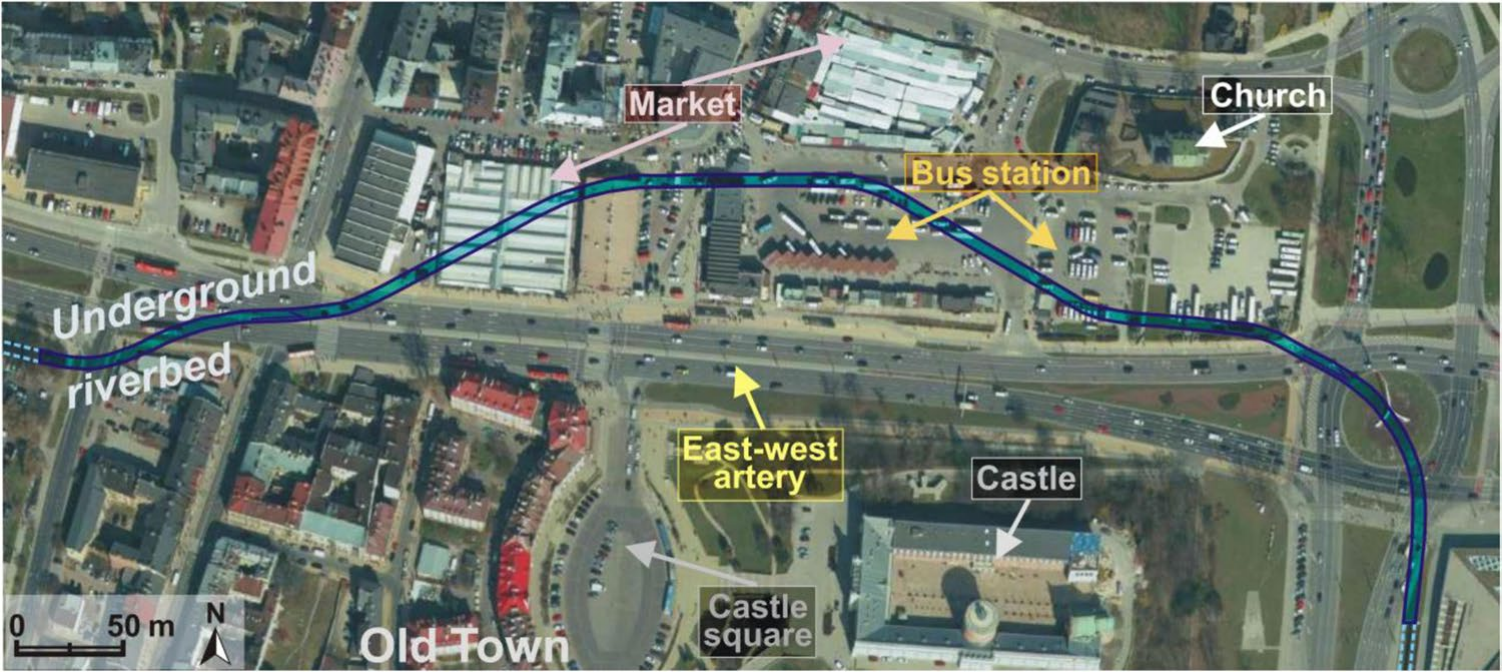
The contemporary course of the underground river bed including the use of adjacent grounds

In 1937, in the downstream section of the valley, the river bed was covered with a concrete ceiling and converted into an underground channel about 0.8 km long (Figs. 3 and 4). On the one hand, this procedure resulted in the availability of a large area for the construction of a central bus station, and on the other hand, contributed to a complete modification of the hydrological regime of the valley.

**Fig. 5:**
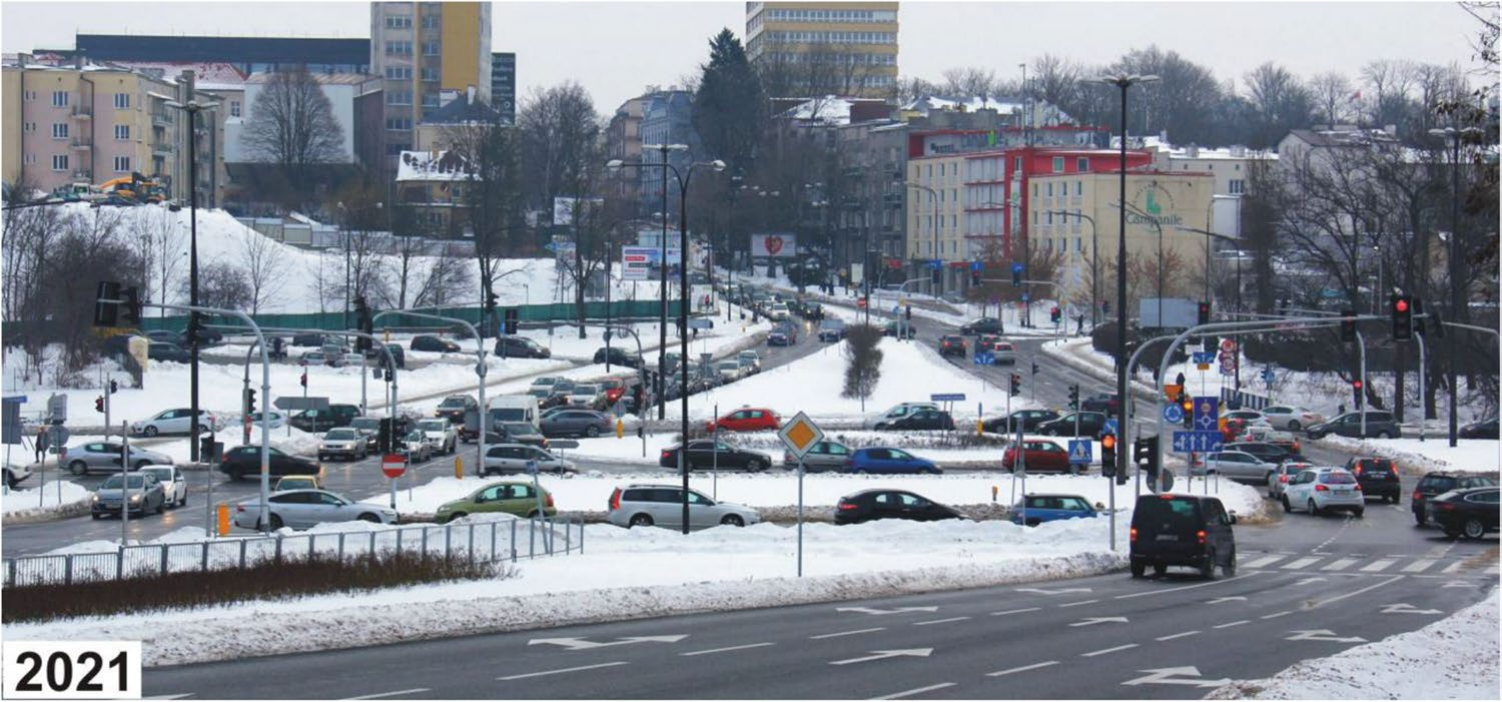
The location of the non-existent water reservoir visible on the maps from 1829 and 1884

The use of underground waters intensified. In 1961, the construction of a water intake with nine wells in the middle of the valley’s length contributed to reducing the river flows. Due to a change in the hydraulic balance, surface water infiltrated into the underground resources (Michalczyk et al. 2017). The water reservoir with a surface area of about 10 ha was transformed into one of the largest transport hubs in the city (Figs. 2 and 5).

In the lower section of the valley, the residential buildings destroyed during the World War II were replaced with the Castle Square, a large city marketplace and a further section of the east–west transit road (national and international road functions) (Fig. 4). As a consequence, not only were the creations of nature transformed but the centuries-long human material legacy—an important complement to the Old Town existing until this day—was obviated. The area was almost completely sealed (disturbance of rainwater infiltration). For the then planners, the concept of building a two-lane transport artery on a flat and wide bottom with long straight-line sections was a very comfortable solution. Even a several metres thick peat base was not a barrier to project performance. The transit road with 2–4 lanes in each direction efficiently separates the inhabitants of the left side of the valley from the river. After the road was built, the share of water-tight surfaces in the bottom of the valley considerably increased, which reduced the natural water retention capacity of the valley (the sealed surface covers about 65% of the bottom of the analysed section). Such measures seriously deform the local water cycle (Geiger and Dreiseitl 2001; Patro and Zubala 2020). Natural vegetation was replaced with roadside lawns. A few old poplars and willows survived as relics of the natural riverside riparian forests. The previous valley microclimate (humid in the flood plain and warm on the sunny slopes) changed into a valley city microclimate with considerable air pollution and very unfavourable acoustic conditions (Geoportal Miejski 2020). Most of the side valleys were turned into streets and thus the natural terrain relief was considerably transformed. The number of transverse roads intersecting the bottom of the valley and thus the number of bridges increased. Buildings were constructed on many slopes (Fig. 2). The same applies to certain fragments of the bottom considered flood plains. A considerable part of them is occupied by car parks. The space of the valley ceased to be a naturally functioning landscape arrangement. The strong transformation of the valley and deterioration of river water quality decreased the attractiveness of this area to the inhabitants. The current property status is characterized by a large share of land and real property owned by the municipality and the State Treasury (Geoportal Miejski 2020).

**Fig. 6:**
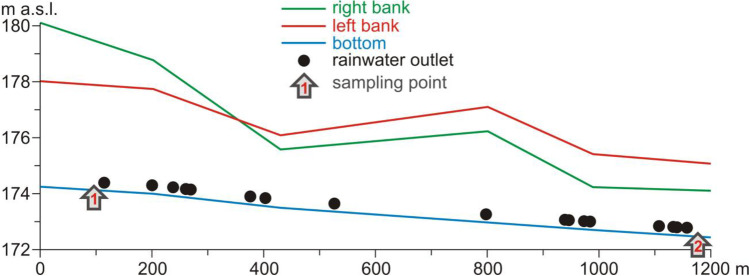
Longitudinal section of the bed of the Czechówka River in the part covered by detailed analyses

During the construction of the transport artery in the 1970s, nearly 4 km of the river bed was dug through. The construction of an extension of the arterial road to the new hub in 2014 resulted in the destruction of the last natural fragment of the Czechówka River near the western boundary of the city. The picturesque meanders with adjacent wet meadows and ponds were backfilled and the river was routed in a straight-line concrete channel. The river bed on the section subject to detailed analyses has exceptionally unnatural parameters and appearance. It is reminiscent of a deep cut canal with steep banks, narrow bottom and a small amount of flowing water. The longitudinal slope of the bottom is 0.16%, and its width ranges from 1.7 to 2.9 m. The bed is from 1.8 to 3.7 m deep, and the average bank slope is 24.1–54.2%. Along 1200 m of the river bed, 17 stormwater outlets in total were placed on both sides (Fig. 6). They drain, for instance, stormwater runoffs from the traffic artery. The outlets are not equipped with stormwater pre-treatment devices.

### The impact of urbanization on river water flows

In 2013–2016, the average water flow intensity at checkpoint 2 reached 0.27 m^3^⋅s^−1^. The extreme flow rates in the measurement section were 0.09 and 1.22 m^3^⋅s^−1^, whereas water levels ranged from 0.19 to 0.73 m (on average 0.32 m). The rises in water level were short-term and were caused, among other factors, by stormwater discharge through sewage outlets. Over the years, the water flow intensity in the river was observed to decrease. In 2016, the mean flow rate was almost three time lower than in 2013. According to Michalczyk et al. (2017), hydrogeological changes lead to a gradual reduction in the water level and flow in the rivers of Lublin. Hydrological measurements should be continued in the future in order to evaluate the risk of a perennial river being transformed into an intermittent river.

The analysis of scientific papers and hydrographic maps showed that the consumption of water in the city for municipal and industrial needs had been increasing in the latest decades (Michalczyk et al. 1983, 2017; Michalczyk and Łoś 1997). It resulted from the dynamic demographic and economic development. Due to limited available surface water resources, the requirement of water is covered using underground resources. However, an intense intake of underground water has negative hydrogeological consequences. The water table is observed to lower and a depression cone is formed. This phenomenon is also associated with a highly variable atmospheric supply and the progressing land surface sealing, including river valley bottoms being covered with transport infrastructure (reducing of infiltration and valley retention). The largest range of the depression cone in Lublin was observed in 1992 and was 201 km^2^ (water intake 50 million m^3^⋅year^−1^). The water table level dropped by several metres at that time. Figure 1 presents the range of the depression cone according to the analogue hydrographic map from 2007.

### The impact of traffic intensity on the quality of river water

The results of analyses of river water point to a moderate differentiation in its quality. The coefficient of variation exceeded 100% only for suspended solids and amounted to about 255%. For nearly half of water quality ratios, the variation was lower than 50%. The lowest value of this parameter was recorded for pH. The variables that considerably deteriorated the quality of runoff water were suspended solids, NO_2_^−^, PO_4_^3−^ and Fe^3+^. The mean concentration of those pollutants amounted to 71.30, 0.24, 0.51 and 0.67 mg⋅dm^−3^ respectively. Considering the above-given values and the average water flow intensity (0.27 m^3^⋅s^−1^), the annual pollutant load discharged from the catchment basin could reach 607.10 (suspended solids), 2.04 (NO_2_^−^), 4.34 (PO_4_^3−^) and 5.71 Mg (Fe^3+^).

**Fig. 7:**
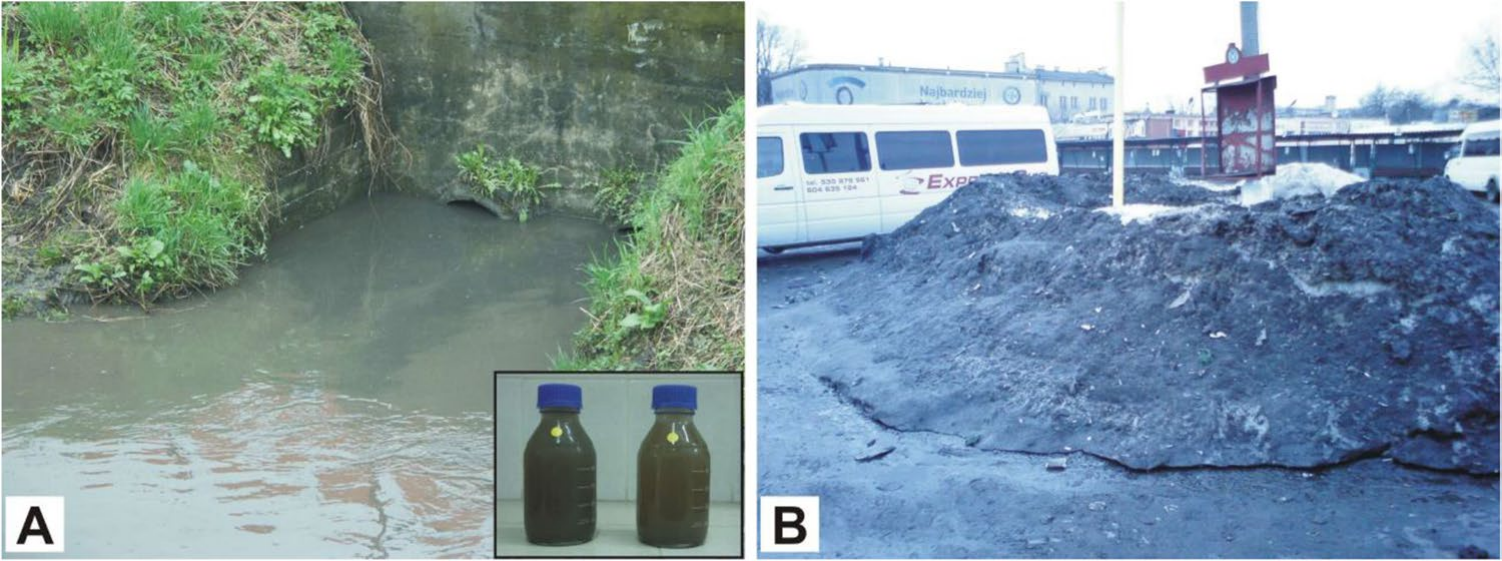
Sources of pollution of the Czechówka River. **A** Discharge of storm water (outlets of the drainage system). **B** Outflow of meltwater (contaminated snow piles at the bus station)

Suspended solid content was especially alarming in the study period as it reached up to 755 mg⋅dm^−3^. Suspended solids are typical pollutants in stormwater running off traffic routes and urbanized areas. They can carry many other dangerous pollutants, e.g. heavy metals or nutrients (Wang et al. 2021; Wu et al. 2018). The content of some pollutants increased in the Czechówka River after intense rainfall and snow melt (e.g. suspended solids, conductivity, COD, some nutrients and chlorides) (Fig. 7).

The studies described water quality characteristics in the study years for heavy and low motor traffic within the traffic artery running along the Czechówka River. According to data provided by the Road and Bridge Administration in Lublin (2019), the mean traffic intensity in 2014 (before the construction of the city’s outer ring road) amounted to 8675 vehicles at peak hours (3:00 PM-5:00 PM), whereas in 2016 (after the construction of the ring road), this had dropped to 5932 vehicles (32% reduction). The share of trucks in the stream of motor vehicles considerably decreased. Taking the percentage differences in the mean values of quality variables, a reduction in the level of pollution in the Czechówka River could be observed for most of the analysed parameters. However, statistical analyses showed significant differences only for the values of conductivity, suspended solids, NO_3_^−^, NO_2_^−^ and K^+^. The highest drop in the concentration was recorded for suspended solids—as much as 91.5%. Except for NH_4_^+^ and PO_4_^3−^ the content of which remained almost identical in both periods, the content of other nutrients fell within the range of 23.3–39.0% (Table 1). The new bypass took over not only a significant part of vehicle traffic, but also related pollution (Zubala and Patro 2021).

**Table 1:** Characteristic values of the fundamental indicators of river water quality in the period with heavy (1: 2013–2014) and low (2: 2015–2016) motor traffic intensity (statistical significance of differences in quality variables was determined using the Mann–Whitney test for *α* = 0.1)

Variables	Period	Minimal value	Maximum value	Average	Median	Standard deviation	Variation coefficient	Important difference
Temperature (°C)	1	2.5	21.0	11.4	11.3	6.9	60.6	−
	2	3.5	17.0	10.3	11.0	5.4	52.0	
Conductivity (μS⋅cm^−1^)	1	233	891	720	748	160.2	22.3	+
	2	210	791	643	720	182.3	28.4	
pH	1	7.4	8.0	7.7	7.8	0.2	2.1	−
	2	7.1	8.0	7.6	7.6	0.3	3.4	
Suspension (mg⋅dm^−3^)	1	3	755	132	36	242.0	183.9	+
	2	2	39	11	9	10.1	90.0	
O_2_ (mg⋅dm^−3^)	1	5.6	12.7	9.7	9.9	1.9	20.0	−
	2	4.4	12.4	8.9	8.9	2.4	27.1	
BOD _5_ (mg⋅dm^−3^)	1	1.4	6.7	3.1	2.8	1.5	48.8	−
	2	0.1	5.1	2.9	3.2	1.6	55.9	
COD_Cr_ (mg⋅dm^−3^)	1	3	22	11	13	5.4	48.9	−
	2	2	48	17	14	11.7	67.8	
NH_4_^+^ (mg⋅dm^−3^)	1	0.10	0.56	0.27	0.26	0.1	50.5	−
	2	0.10	0.95	0.26	0.17	0.3	99.5	
NO_3_^−^ (mg⋅dm^−3^)	1	1.04	8.61	4.25	4.73	2.4	56.0	+
	2	0.51	4.65	2.59	2.60	1.1	41.7	
NO_2_^−^ (mg⋅dm^−3^)	1	0.14	1.10	0.28	0.21	0.23	82.6	+
	2	0.10	0.58	0.20	0.15	0.15	72.6	
PO_4_^3−^ (mg⋅dm^−3^)	1	0.01	0.74	0.51	0.57	0.2	39.3	−
	2	0.22	0.79	0.52	0.52	0.1	27.8	
SO_4_^2−^ (mg⋅dm^−3^)	1	14	179	96	103	43.3	45.3	−
	2	4	147	98	117	47.1	48.2	
Fe^3+^ (mg⋅dm^−3^)	1	0.22	1.49	0.76	0.65	0.4	51.5	−
	2	0.26	1.08	0.58	0.58	0.2	34.4	
K^+^ (mg⋅dm^−3^)	1	4.2	20.6	12.1	11.9	5.5	45.2	+
	2	2.9	19.2	8.7	6.5	5.8	66.2	
Cl^−^ (mg⋅dm^−3^)	1	9.5	28.1	21.8	23.3	4.8	21.8	−
	2	7.6	27.9	21.3	23.2	6.7	31.3	

The water pollution levels in the Czechówka River at checkpoints 1 and 2 were also compared (level of significance *α* = 0.1). Significant differences in the distribution of the analysed characteristics were found for conductivity, BOD_5_, NO_3_^−^, SO_4_^2−^, K^+^, Cl^−^ (increased down the river) and PO_4_^3−^ (decreased down the river). Water was more polluted at checkpoint 2 than at checkpoint 1 (accumulation of pollutants), whereas the differences between the mean values of the above-mentioned variables were not large and ranged from 4.3 (conductivity) to 15.2% (SO_4_^2−^). At most measurement dates, the level of suspended solids was similar at both checkpoints (on average 72 and 71 mg⋅dm^−3^).

A large differentiation in the quality of river water was observed in the cold season (autumn, winter) and the warm season (spring, summer). In each of these periods, different weather conditions were observed (atmospheric precipitation, ambient temperature, snow cover, etc.) (SP 2018). In the warm season, 80% of the analysed indicators were more varied than in the cold season. In particular, this referred to suspended solids, BOD_5_, COD, NH_4_^+^, NO_2_^−^, SO_4_^2−^, Fe^3+^ and K^+^. In the warm season, the mean water temperature increased by 207% (mean temp. 16.4 °C). A significant increase was also observed in the values of COD, NO_2_^−^ and PO_4_^3−^ (*α* = 0.1). On the other hand, lower values were noted for conductivity, O_2_, SO_4_^2−^, K^+^ and Cl^−^. Elevated values of electrolytic conductivity and Cl^−^ in river water in the cold season testify to the supply of meltwater containing, among other pollutants, salt used to reduce slipperiness after snowfall (Ociepa et al. 2015).

The direct supply of polluted precipitation water to river bed sections situated in the city becomes a globally increasing problem. Rainwater captures all pollutants it finds on its way—both in the air and on sealed surfaces (such as roads, car parks, fairgrounds) (Beck and Birch 2012; Zubala 2018, 2019). Limited retention results in extremely high and polluted surface runoffs, which in turn can induce hydrobiological stress in water courses (impact of harmful physical, chemical and biological factors) (Geiger and Dreiseitl 2001). These phenomena are random and they have not been satisfactorily examined yet.

### The concept of revitalisation of the valley passing through the city centre

Despite the progressing urbanisation of the river valley, some sections can still be revitalized, which is fostered by transferring a significant part of motor traffic to areas outside the city and by the planned liquidation of the central bus station. The spatial structure of the analysed area consists of many architectural and landscape units that, following re-composition and natural enrichment, should form the main structural elements of an attractive complex with natural, social and economic functions. Two sub-sections have been selected in the river valley—with an open river bed and with an underground river bed.

The plans to revitalize the valley with an open river bed raise no significant objections. Stakeholders agree to increase the share of green areas and recreational and leisure facilities. According to the author, it is necessary to supplement the trees and shrubs growing along the river course. Creeks, meanders and small ponds must be formed in the river bed. Since the river bed is quite deep, a cascade of stone dams can be built to have an advantageous effect on water resources both in terms of quantity (elevated level of surface waters and groundwaters, stabilisation of flows) (Mioduszewski et al. 2014) and quality (sedimentation, filtration and oxygenation—particularly important in case of decreasing the mean annual content of oxygen in water) (Imhoff and Imhoff 2006). The quality of surface water can also be improved by installing pre-treatment devices at the stormwater sewerage outlets (e.g. settling tanks and separators) (Daligault et al. 1999; Tran and Kang 2013). Wherever possible, the surface must be unsealed in order to increase the infiltration of rainwater and enhance the resources of underground water (combating the depression cone). On both sides of the river, pedestrian and cycling routes must be built connected with bridges (the main natural and leisure axis). The imperative objectives of revitalisation include maintaining high landscape values of the loess steep slopes (Song et al. 2012). It is necessary to separate the car parks on the bottom of the valley with belts of masking vegetation and improve their surface and use greenery in the internal arrangement. Adequate pavement permeability must be ensured. It is recommended to create a lane of trees and shrubs on the side of the traffic artery, using species characteristic for river valleys. Such a solution would also partially prevent air pollution (Morakinyo and Lam 2016) and transport noise (Fang and Ling 2003) and would have a beneficial effect on the microclimate (Yilmaz et al. 2021). The presented proposals will enhance the natural potential of the discussed section of the valley and extend its recreation and leisure functions (Keeley and Benton-Short 2019; Säumel et al. 2016).

**Fig. 8:**
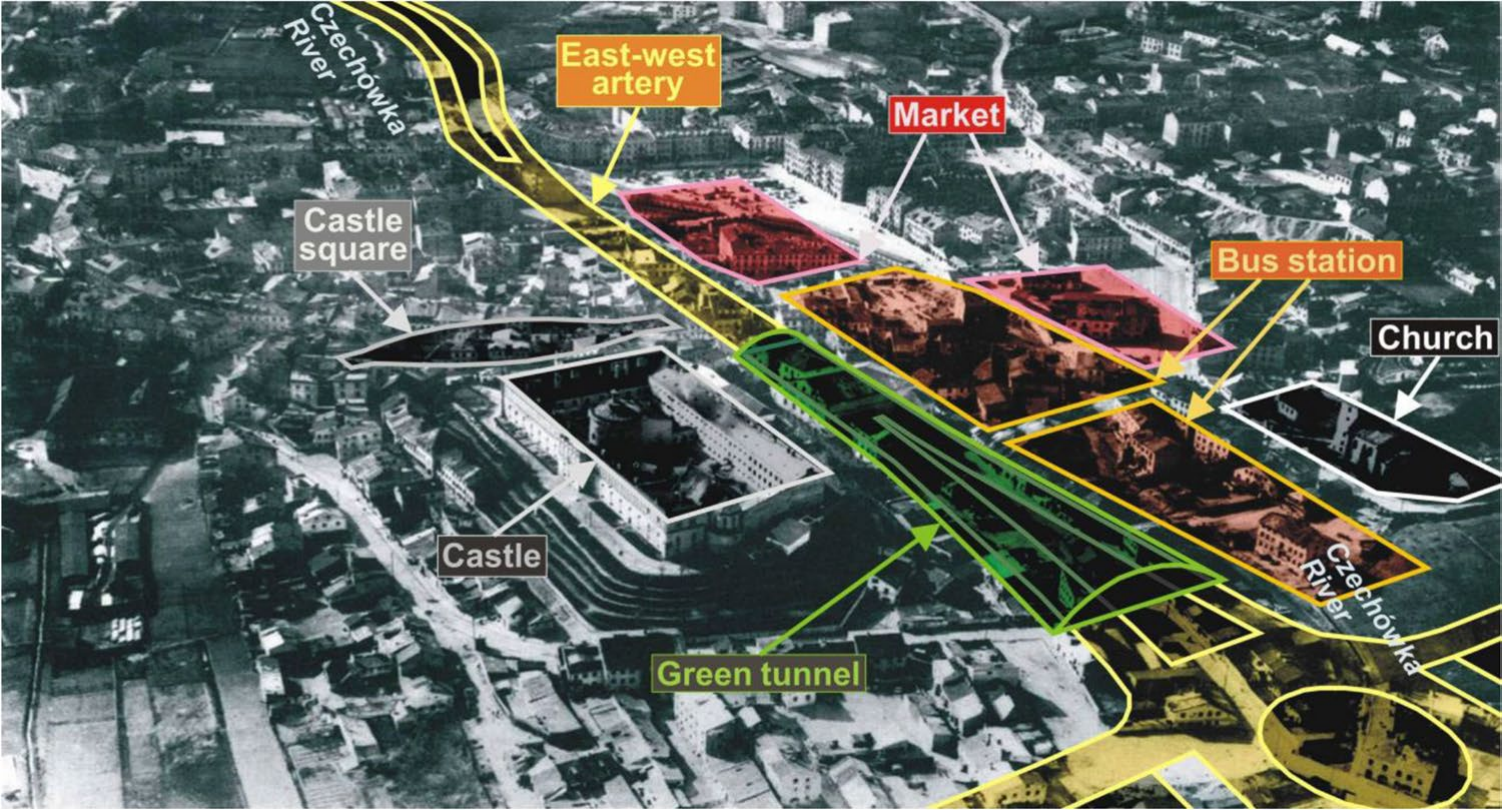
The estuarine section of the Czechówka River (1930s) (Grodzka Gate – NN Theatre 2019); shaded areas mark the present functions of the grounds and the proposed green tunnel

The greatest doubts concern the management plans of the estuarine section of the valley with the river turned into an underground canal (Fig. 4). In the author’s opinion, each concept of its revitalisation should start with uncovering the river bed. Water is a very important element of urban architecture. It allows maintaining contact with nature and is a strong symbol of not only evanescence but also purification. Blue space has a positive effect on public health (Völker et al. 2016; Well and Ludwig 2020). Uncovering the river and adequate management of the adjacent grounds would allow creating an attractive space in the very centre of the city. Trends in contemporary public space design are oriented at restoring the old terrain relief and emphasizing the local natural resources (Đurakovac 2016; Temperton et al. 2014). Exhibiting the river would create a space making reference to the historical terrain relief in the city and become an unquestionable amenity to inhabitants and tourists. The urban planning solutions dating back to the 1930s (the heyday of the city) must be partly reconstructed—among other things, the compact, structured building development (living quarters, hotels, small restaurants and shops, underground car parks) with narrow streets, small squares and plazas should be restored. As a modern solution, new buildings should be covered with green roofs, which would ensure the continuity of the existing and projected natural objects in the valley of the Czechówka River as well as partly retain and pre-treat the precipitation waters (including reduction of transport pollution) (Vijayaraghavan et al. 2019; Yao et al. 2020). Green roofs would be very visible from the Castle Hill and other vantage points. A good solution would be a green tunnel over the traffic artery after its level is lowered (length 200 m) (Fig. 8).

The tunnel would isolate the road, at the same time maintaining a view of the Castle Hill. It would form an integral whole with the adjacent grounds, including the commons on the other side of the castle. The level of pollution would be limited thanks to the use of adequate adsorption materials inside and the level of noise would be reduced (Kim et al 2019; Ramirez et al. 2010). The outer surface of the tunnel could be used as an overpass above the traffic artery and become a meeting and entertainment place for the inhabitants (beach in the city centre).

The concept also provides for education and information on the changes in the natural character of the valley. In the places where the old functions cannot be reconstructed, it is proposed that the location of the former land and objects be indicated. An example can be low vegetation (a hedge, decorative grass) delineating the limits of the non-existent ponds. Technical means such as, for example, the routing of the old river bed marked on the surface of the traffic artery and pedestrian and cycling routes are also worth using. The green tunnel is also an excellent place to display pieces of information about the history associated with the oldest part of the city that is located nearby. These activities can help to better understand the processes of environmental change, their effects and human participation.

### Conflict of interest and urban succession

The largest problem is posed by making a decision on the directions and plan of management of the estuarine section of river valley after the bus station and marketplaces are closed. This is where different social and economic interests clash. A number of concepts have been put forward, while the most viable proposal is the one that primarily takes into account economic aspects (e.g. commercial and hotel buildings, car parks, leaving the river underground). This concept ignores most of the needs and the hidden social and natural potential of the analysed space.

In view of the pending disputes and controversies regarding the choice of function and method of management of the estuarine section of the river valley, this paper proposes to make use of solutions existing in the natural environment and introduce *urban succession and urban climax* as new terms and criteria helping make important decisions. The terms succession and climax have been long used in ecology (Odum and Barrett 2004). The first of them means an ordered, gradual process of directional changes and consequences leading to the transformation of simple ecological systems into more complex ones. In natural conditions, such changes result in transforming the space and the environment and developing a new ecological system characterized by a state of balance and self-sufficiency—the climax. The climax is simultaneously a reflection of all the past phenomena. These can include the entry of pioneer organisms to an uninhabited land, migrations, settlement in all unoccupied spaces, competition and increased complexity of different relationships, stabilisation with relative balance between the biotic and abiotic elements of the space (homeostasis). At the climax stage, the needs of all biotic components are satisfied and the environment is not at risk of destruction. The definitions of *urban succession* and *urban climax* must similarly emphasize the condition of the lack of impact causing the degradation of the environment and society as well as consider the historical and cultural changes (accumulated transformations) as important factors that govern the functioning of the specific space. According to the author, such an order was captured in the photo from the 1930s (Fig. 8).

The order and self-sufficiency should be attained with the participation of, among other agents, the local community, planners, landscape architects, officials and investors (Kitheka et al. 2021; Martens and van Weelden 2013). In addition, the needs of the largest possible group of the parties concerned should be satisfied. In this context, reconstructing the old part of the lower old town with all the necessary services and housing estates, introducing greenery, uncovering the river, building a green tunnel above the traffic artery and at the same time maintaining this artery seem the right solution (Fig. 8). This will be a return to the verified concept and the legacy of the ancestors deriving from centuries-long succession processes and respecting the best effects of contemporary transformations (Montalbán Pozas and Neila González 2018).

## Conclusions

The studies showed considerable changes in the management and use of the river valley section located in the city centre that have occurred over the past two centuries, which was a result of the need to satisfy the basic needs of local communities and improve the living conditions within the general meaning of this term. The increasingly rapid observed development of the social and economic system took place at the expense of an intensely used natural system. Transformation and destruction affected mainly land relief, water and meadow ecosystems, which to a large extent was connected with the emergence of transport infrastructure. Not only did the changes in the management and use of the valley area transform the creations of nature but also the centuries-long material legacy of man.

Due to the absence of an assessment of the impact of spatial development plans on the environment in making decisions related to spatial planning, the landscape values and water resources considerably deteriorated. The liquidation of objects performing natural functions and the sealing of land surface (limited infiltration), with a simultaneous intensification of the intake of underground water for municipal and household purposes, led to the formation of a huge depression cone underneath the urban area, including within the analysed river valley. The process was linked to decreasing water levels and flows in rivers. Accumulated negative environmental impacts can be prevented exclusively by way of collaboration between specialists in different fields undertaken at the project preparation stage.

Untreated rainwater from the traffic artery was discharged into the analysed river that the then decision-makers considered the safest and the cheapest receiver. This deepened its degradation. As a result of strong transformation and simplification of the valley area and the deterioration of the quality of water in the river, these grounds became less attractive. The quality of water in the river was observed to improve after transferring a considerable part of motor traffic from the artery passing through the city centre to the new outer ring road of the city. The values of the most problematic indicators—electrolytic conductivity, suspended solids and most nutrients—dropped. Such decisions and their beneficial consequences should precede the process of revitalisation of the river valleys passing through the city centres with a well-developed transport infrastructure within its limits.

In the case of transformed urban valleys, the best solution would be to restore the old functions and management, taking into account some solutions of present-day urban planning trends. The concept of revitalisation should involve the necessity of uncovering the river bed. The river must be considered the most important resource and a characteristic element of the city centre and restructuring of the analysed space should start from there. Revitalisation should cover both natural enrichment of ecosystems and extension of the recreation and leisure, housing and service functions based on the wealth of nature, harmony between landscape and architecture, and cultural heritage and tradition determined by centuries-long urban succession processes.

## Data Availability

All data generated or analysed during this study are included in this published article.

## References

[CR1] Angelidou M, Psaltoglou A (2017). An empirical investigation of social innovation initiatives for sustainable urban development. Sustain Cities Soc.

[CR2] Arslan G, Gultekin AB, Kivrak S, Yildiz S (2020). Built environment design - social sustainability relation in urban renewal. Sustain Cities Soc.

[CR3] Beck HJ, Birch GF (2012). Metals, nutrients and total suspended solids discharged during different flow conditions in highly urbanised catchments. Environ Monit Assess.

[CR4] Chan KM, Vu TT (2017). A landscape ecological perspective of the impacts of urbanization on urban green spaces in the Klang Valley. Appl Geogr.

[CR5] Chmielewski TJ (2005) Principles of cities’ ecological revitalization planning and its accomplishment management. Teka Komisji Architektury, Urbanistyki i Studiów Krajobrazowych, 60–67. (in Polish)

[CR6] Daligault A, Meaudre D, Arnault D, Due V, Bardin N, Aires N, Biau D, Schmid J, Clement P, Viau J-Y (1999). Stormwater and lamella settlers: efficiency and reality. Water Sci Technol.

[CR7] Đurakovac A (2016) What makes Roombeek the brook a remarkable urban street? Land8: Landscape Architects Network. https://land8.com. Accessed 4 May 2020.

[CR8] Fang C-F, Ling D-L (2003). Investigation of the noise reduction provided by tree belts. Landsc Urban Plan.

[CR9] Frantzeskaki N (2019). Seven lessons for planning nature-based solutions in cities. Environ Sci Policy.

[CR10] Geoportal Miejski (2020) Lublin Spatial Information System. https://geoportal.lublin.eu. Accessed 10 April 2019.

[CR11] Geiger W, Dreiseitl H (2001) New methods of rainwater drainage. Oldenbourg Verlag, München (in German)

[CR12] Grodzka Gate – NN Theatre (2019) Multimedia library. http://biblioteka.teatrnn.pl. Accessed 7 May 2019.

[CR13] Imhoff K, Imhoff KR (2006) Handbook of urban drainage. Oldenbourg Industrieverlag, München (in German)

[CR14] Keeley M, Benton-Short L (2019). Urban green space. Urban Sustainability in the US.

[CR15] Kim YH, Song GG, Park JH, Kim SY, Lee SC (2019) Acoustic characteristics of tunnel-shaped noise barriers. INTER-NOISE and NOISE-CON Congress and Conference Proceedings, Inter Noise 2019, June 16–19, Madrid, Spain, 6152–6157.

[CR16] Kitheka BM, Baldwin ED, Powell RB (2021). Grey to green: tracing the path to environmental transformation and regeneration of a major industrial city. Cities.

[CR17] Kociuba D (2003) Changes in the functions of river valleys in the Lublin area. Annales Universitatis Mariae Curie-Skłodowska Lublin, LVII I(5):121–137 (in Polish)

[CR18] Li L, Lei Y, Wu S, He C, Chen J, Yan D (2017). Optimal scale of China’s cities under the maximization of economic benefits and environmental benefits. Environ Sci Pollut Res.

[CR19] Luo T, Zhang T, Wang Z, Gan Y (2015). Driving forces of landscape fragmentation due to urban transportation networks: lessons from Fujian, China. Journal of Urban Planning and Development.

[CR20] MAPSTER (2019) Archival maps of Poland and Central Europe. http://igrek.amzp.pl. Accessed 19 June 2019.

[CR21] Martens K, van Weelden P (2013). Decision-making on transport infrastructure and contested information: a critical analysis of three approaches. Eur Plan Stud.

[CR22] Michalczyk Z, Łoś MJ (1997). Anthropogenic changes in water conditions in the Lublin area. Geogr Pol.

[CR23] Michalczyk Z, Chmiel S, Głowacki S, Sposób J (2017) Exploitation of groundwaters resources in Lublin in 1955–2015. Przegląd Geologiczny 65(11/2):1344–1349 (in Polish)

[CR24] Michalczyk Z, Łoś M, Sawicka-Ner Z (1983) The range of the impact of underground water intakes in Lublin. Inst. Geol, Warsaw. (in Polish)

[CR25] Ministry of Infrastructure (2020) Program for the construction of 100 ring roads. https://www.gov.pl. Accessed 10 April 2020.

[CR26] Mioduszewski W, Querner EP, Kowalewski Z (2014). The analysis of the impact of small retention on water resources in the catchment. Journal of Water and Land Development.

[CR27] Montalbán Pozas B, Neila González FJ (2018) Housing building typology definition in a historical area based on a case study: the Valley, Spain. Cities 72(A):1–7. 10.1016/j.cities.2017.07.020

[CR28] Morakinyo TE, Lam YF (2016). Study of traffic-related pollutant removal from street canyon with trees: dispersion and deposition perspective. Environ Sci Pollut Res.

[CR29] Ociepa E, Mrowiec M, Deska I, Okoniewska E (2015) Snow cover as a medium for deposition of pollution. Rocznik Ochrona Środowiska 17:560–575 (in Polish)

[CR30] Odum EP, Barrett GW (2004). Fundamentals of ecology.

[CR31] Patro M, Zubala T (2020) Use of different forms of retention as the condition of sustainable management of water resources in rural environment. Journal of Water and Land Development 44(I-II):126–135. 10.24425/jwld.2019.127053

[CR32] Peng B, Sheng X, Wei G (2020). Does environmental protection promote economic development? From the perspective of coupling coordination between environmental protection and economic development. Environ Sci Pollut Res.

[CR33] Pincetl S (2012). Nature, urban development and sustainability – what new elements are needed for a more comprehensive understanding? Cities 29. Supplement.

[CR34] Ramirez AM, Demeestere K, De Belie N, Mäntylä T, Levänen E (2010). Titanium dioxide coated cementitious materials for air purifying purposes: preparation, characterization and toluene removal potential. Build Environ.

[CR35] Ren C, Yang R, Cheng C, Xing P, Fang X, Zhang S, Wang H, Shi Y, Zhang X, Kwok YT, Ng E (2018). Creating breathing cities by adopting urban ventilation assessment and wind corridor plan – the implementation in Chinese cities. J Wind Eng Ind Aerodyn.

[CR36] Road and Bridge Administration in Lublin (2019) Road traffic measurements. http://www.zdm.lublin.eu. Accessed 11 May 2019.

[CR37] Säumel I, Weber F, Kowarik I (2016). Toward livable and healthy urban streets: roadside vegetation provides ecosystem services where people live and move. Environ Sci Policy.

[CR38] SO (Statistical Office) (2019) Lublin City. Statystyczne Vademecum Samorządowca, Lublin. (in Polish)

[CR39] Song W, Yongguo Z, Xiaoxu G, Xiaoning L (2012) Slope landscape classification and application security in the special section of Western. Procedia Environmental Sciences 12(A):146–151. 10.1016/j.proenv.2012.01.259

[CR40] SP (Statistics Poland) (2018) Environment 2018. Statistical Analyses, Warsaw.

[CR41] Suárez M, Barton DN, Cimburova Z, Rusch GM, Gómez-Baggethun E, Onaindia M (2020). Environmental justice and outdoor recreation opportunities: a spatially explicit assessment in Oslo metropolitan area, Norway. Environ Sci Policy.

[CR42] Tarolli P, Vanacker V, Middelkoop H, Brown AG (2014). Landscapes in the Anthropocene: state of the art and future directions. Anthropocene.

[CR43] Temperton VM, Higgs E, Choi YD, Allen E, Lamb D, Lee C-S, Harris J, Hobbs RJ, Zedler JB (2014). Flexible and adaptable restoration: an example from South Korea. Restor Ecol.

[CR44] Tran D, Kang JH (2013). Optimal design of a hydrodynamic separator for treating runoff from roadways. J Environ Manage.

[CR45] Vijayaraghavan K, Harikishore Kumar Reddy D, Yun Y-S (2019). Improving the quality of runoff from green roofs through synergistic biosorption and phytoremediation techniques: a review. Sustain Cities Soc.

[CR46] Virág D, Wiedenhofer D, Haas W, Haberl H, Kalt G, Krausmann F (2021) The stock-flow-service nexus of personal mobility in an urban context: Vienna Austria Environmental Development 100628. 10.1016/j.envdev.2021.100628

[CR47] Völker S, Matros J, Claßen T (2016). Determining urban open spaces for health-related appropriations: a qualitative analysis on the significance of blue space. Environmental Earth Sciences.

[CR48] Wang J, Qin M, Huang T, Tu N, Li B (2021). Particle size distribution and pollutant dissolution characteristics of road-deposited sediment in different land-use districts: a case study of Beijing. Environ Sci Pollut Res.

[CR49] Weaver KF, Morales V, Dunn SL, Godde K, Weaver PF (2017). An introduction to statistical analysis in research: with applications in the biological and life sciences.

[CR50] Well F, Ludwig F (2020). Blue–green architecture: a case study analysis considering the synergetic effects of water and vegetation. Frontiers of Architectural Research.

[CR51] White RR (2002). Building the ecological city.

[CR52] Wu X, Yu J, Qiu H, Fang H (2018). Pollution and ecological risk assessment of nutrients associated with deposited sediments collected from roof and road surfaces. Environ Sci Pollut Res.

[CR53] Xue J (2012). Potentials for decoupling housing-related environmental impacts from economic growth. Environmental Development.

[CR54] Yao L, Wu Z, Wang Y, Sun S, Wei W, Xu Y (2020). Does the spatial location of green roofs affects runoff mitigation in small urbanized catchments?. J Environ Manage.

[CR55] Yilmaz S, Mutlu BE, Aksu A, Mutlu E, Qaid A (2021). Street design scenarios using vegetation for sustainable thermal comfort in Erzurum, Turkey. Environ Sci Pollut Res.

[CR56] Zoeteman K, Mommaas H, Dagevos J (2016). Are larger cities more sustainable? Lessons from integrated sustainability monitoring in 403 Dutch municipalities. Environmental Development.

[CR57] Zubala T (2018). Technical and natural conditions and operating efficiency of a municipal stormwater treatment plant. Environ Sci Pollut Res.

[CR58] Zubala T (2019). Time and space variability of water quality in the inner-city river in Lublin from the aspect of existing natural and land use conditions. Rocznik Ochrona Środowiska.

[CR59] Zubala T, Patro M (2021). Time and spatial variability in concentrations of selected pollutants in the new bypass rainwater harvesting system. Water Air Soil Pollut.

